# Use of siphon irrigation during burr-hole craniostomy to evacuate chronic subdural hematoma: A retrospective cohort comparison study

**DOI:** 10.1097/MD.0000000000020291

**Published:** 2020-05-22

**Authors:** Song Chen, Zhen Chen, Bin Yang, Tao Xu, Xian-kun Tu

**Affiliations:** Department of Neurosurgery, Fujian Medical University Union Hospital, Fuzhou, Fujian, China.

**Keywords:** burr-Hole craniotomy, chronic subdural hematoma, outcome, siphon irrigation

## Abstract

Burr-hole craniostomy (BHC) is a widely accepted treatment for chronic subdural hematomas (CSDHs). This study adopted siphon irrigation to evacuate CSDHs and investigated its efficacy and safety as compared with the traditional irrigation used in BHC.

A retrospective cohort study was conducted at a center between January 2017 and December 2018. The data of 171 patients who underwent burr-hole craniostomy for CSDH were collected and analyzed. A total of 68 patients underwent siphon irrigation (siphon group) and 103 patients were treated by a traditional method (control group). A follow-up was conducted 6 months after the surgery.

No significant difference was observed in the baseline characteristics and preoperative computed tomography (CT) features of the 2 groups (*P* > .05). The postoperative CT features of the siphon group, which included the volume of hematoma evacuation (*P* = .034), hematoma evacuation rate (*P* < .001), recovery rate of the midline shift (*P* = .017), and occurrence of pneumocephalus (*P* = .037) were significantly different and better than those of the control group. The length of hospital stay after surgery of the siphon group was significantly shorter than that of the control group (*P* = .015). The Markwalder score of the siphon group was significantly superior to that of the control group on postoperative day 1 (*P* = .006). Although the recurrence rate in the siphon group (2/68, 2.5%) was lower than that in the control group (11/103, 8.9%), no statistically significant difference was observed between them (*P* = .069). Moreover, no significant differences were observed in terms of complications and mortality rate between the 2 groups.

There was no significant difference in the recurrence rate between the groups that underwent siphon irrigation and traditional irrigation. However, in comparison, siphon irrigation can better improve postoperative CT features, promote early recovery of neurological dysfunction after surgery, and shorten the length of hospital stay. This indicates that siphon irrigation may be a better therapeutic option in BHC for CSDH.

## 1 Introduction

Chronic subdural hematoma (CDSH) is a common disease managed by neurosurgery and it frequently occurs in the elderly.^[1]^ Scholars believe that with the progression of an aging global population and with the increasing use of antiplatelet drugs and anticoagulants, more people will suffer from CDSHs.^[2]^ Surgery is the current treatment for evacuating hematomas, and it usually results in vastly improved neurological function.^[3]^ Three surgical techniques are most frequently used: twist-drill craniostomy, burr-hole craniostomy (BHC), and craniotomy.^[4]^ Among these options, BHC is generally the most preferred technique used by neurosurgeons for the initial treatment of CSDH patients.^[4–6]^

BHC, a classic form of minimally invasive surgery, was popularized by Markwalder and his colleagues in the 1980 s as a viable first-line alternative to craniotomy.^[7]^ Over the years, to further reduce the recurrence rate of CDSH and improve the curative effect, neurosurgeons have devoted themselves to improving the surgical method of BHC. They have focused on various factors, which include the number of drills,^[8]^ whether to perform irrigation,^[9]^ different types of irrigation fluid,^[10]^ different temperatures of irrigation fluid,^[11]^ whether to perform drainage,^[4]^ and the duration of drainage.^[6]^ Certain progress has been achieved. However, only a limited number of studies have focused on the irrigation methods. This retrospective study was conducted to compare the siphon irrigation method with the traditional method to investigate their curative effects on the burr-hole evacuation of CSDHs.

## 2 Materials and methods

### 2.1 Patients

We retrospectively reviewed the medical records of all patients undergoing CSDH surgical treatment at the Department of Neurosurgery of Fujian Medical University Union Hospital between January 2017 and December 2018. The department consists of multiple treatment groups, and these groups receive patients at random according to the date of admission. One treatment group used BHC with siphon irrigation to evacuate CSDHs while the other treatment groups used a traditional method.

The inclusion criteria for the study were as follows:

a) initial unilateral or bilateral CSDH confirmed by computed tomography (CT) and/or magnetic resonance imaging and
b) BHC treatment.

The exclusion criteria were as follows:

a) CSDH recurrence after BHC,
b) bilateral hematoma undergoing a two-stage operation,
c) history of craniocerebral surgery,
d) statin intake before and after surgery, and
e) missing clinical data or patient lost for follow-up.

A bilateral hematoma was defined as one patient with 2 cases of a hematoma.

We collected the medical records of all patients who met the criteria during the above period to avoid the occurrence of patient selection bias. We confirm that any aspect of the work covered in this paper that has involved human patients has been conducted with the ethical approval of Fujian Medical University Union Hospital.

### 2.2 Surgical procedures

The patients’ tolerance level and ability to remain cooperative intraoperatively would be taken into considerations. Thus, the decisions for local or general anesthesia would be made accordingly.

The control group adopted the traditional BHC procedure. A skin incision was made at the thickest layer of the hematoma and was positioned at the highest plane of the head to prevent the occurrence of pneumocephalus. The scalp was sliced through and a 1.2 to 1.5 cm burr hole was drilled. The dura mater was coagulated using bipolar diathermy and opened with a cruciate incision. During the spontaneous drainage of the hematoma, an 8 or 10 mm silastic catheter was placed inside the hematoma cavity and the subdural hematoma was washed repeatedly with normal saline. The position of the silastic catheter was adjusted until the irrigation fluid became almost clear in all directions. Afterward, a subdural drainage tube was indwelled and removed through another scalp incision to avoid infection. A gelatin sponge was placed to fill the bone hole. Finally, the galea aponeurotica and skin were sutured, and the drainage tube was connected to a collection bag.

**Figure 1:**
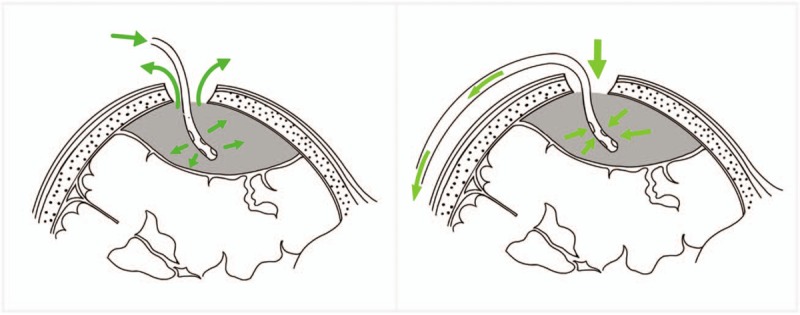
Left: Traditional irrigation. Right: Siphon irrigation.

The surgical treatment of the siphon group differed in the manner of irrigation. When the hematoma spontaneously drained after opening the dura, a saline infusion device was placed at the edge of the incision, and normal saline was continuously injected into the hematoma cavity through the bone hole. Then, the same silastic catheter was inserted into the cavity, and the tail end of the catheter was drooped to make it lower than the hematoma plane. The hematoma was drained through the catheter by the siphon effect. The position of the catheter was adjusted until the fluid ran clear in all directions. Afterward, the drainage tube was retained and the wound was sutured in the same manner as in the control group (Fig. 1).

### 2.3 Postoperative management

A CT scan of each patient's head was taken within 6 to 24 hours after the operation. A subsequent CT scan was performed based on the patient's clinical symptoms and hematoma clearance status. All the patients were in the supine position before the drainage tube was removed. If the patient could assume such a pose, the Trendelenburg position was used. In general, the drainage tube was indwelled for no more than 3 days. If the drainage volume was less than 20 ml in 24 hours, the drainage device was removed in advance. After removal of the drainage device, the patients were encouraged to get out of bed as soon as possible. Conventional prevention of infection and thrombosis was not used after the operation. Patients who had used anticoagulants and antiplatelet drugs before the operation generally resumed their medication 3 to 4 weeks after surgery.

### 2.4 Outcome measures

We collected information regarding the patients’ age, sex, present complaints, trauma history, medical history, drug history, neurological score on admission, coagulation status, anesthesia care, and CT imaging results as baseline data.

The primary outcome measures were recurrence rate and hematoma evacuation rate; the secondary outcome measures included recovery rate of the midline shift, the occurrence of pneumocephalus, drainage volume, duration of drainage, length of postoperative hospitalization, and neurological function on postoperative day 1, time of discharge, and 6 months after surgery.

The recurrence rate was defined as the rate of reoperation to treat a recurrent CSDH in patients who were previously treated with BHC within 6 months. A reoperation was indicated if the original neurological deficit increased, recurred, or did not improve, or if a new neurological deficit arose that needed further surgery—all of which was established by the admitting consultant neurosurgeon.^[4]^

The hematoma evacuation rate was calculated as follows:

Hematoma evacuation rate = (preoperative hematoma volume − postoperative hematoma volume)/preoperative hematoma volume.

Similarly, the recovery rate of the midline shift was calculated as follows:

Recovery rate of the midline shift = (preoperative midline shift − postoperative midline shift)/preoperative midline shift

The hematoma volume and pneumocephalus volume of the CSDH were calculated using the method of XYZ/2.^[12]^ The postoperative hematoma volume, midline shift, and pneumocephalus volume were all obtained from the first CT scan after surgery. To avoid the interference of bilateral surgery, we only analyzed the midline shifts of unilateral hematomas.

An independent neurosurgeon obtained the imaging data from the CT scans, and another neurosurgeon conducted a telephone follow-up 6 months after the surgery to record information regarding neurological function, recurrence, and any patient deaths. The Glasgow Coma Scale and the Markwalder score^[7]^ were used to evaluate the neurological function of patients.

### 2.5 Statistical analysis

All statistical analyses were performed using the Statistical Package for the Social Sciences, version 25.0 (Chicago, IL). Continuous variables were described by mean (±standard deviation) or median (interquartile range (IQR)), and differences between variables were compared using the independent samples t test or Mann–Whitney U test. Categorical variables were described by number (%), and differences between variables were evaluated using the chi-square test or Fisher exact test. The level of significance was set at *P *< .05, and all tests were two-tailed.

## 3 Results

This study included 171 patients with 204 cases of CSDH. The siphon group consisted of 68 patients (54 males, 14 females) and 80 cases, whereas the control group consisted of 103 patients (83 males, 20 females) and 124 cases. There were no significant differences between the two groups in terms of baseline characteristics, preoperative CT features, and anesthesia care (Tables 1–3). A coagulation disorder was absent in both groups. Limb weakness was the most common symptom, followed by headache. The siphon group used more antiplatelet drugs than the control group, but no significant difference was noted between them. No significant difference was observed regarding the type of anesthesia used.

**Table 1:**
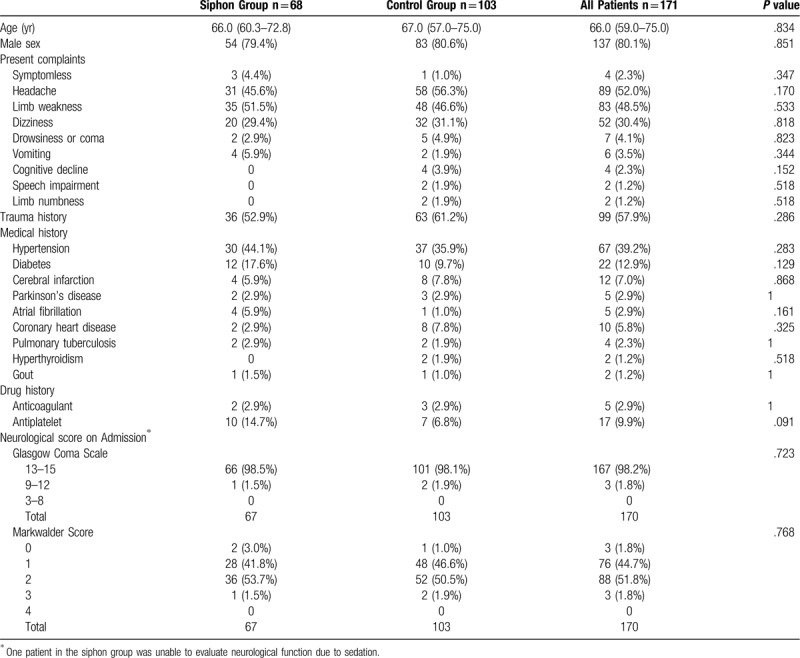
Baseline patient characteristics.

**Table 2:**
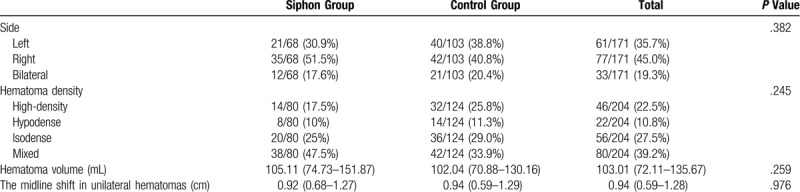
Preoperative CT features.

**Table 3:**
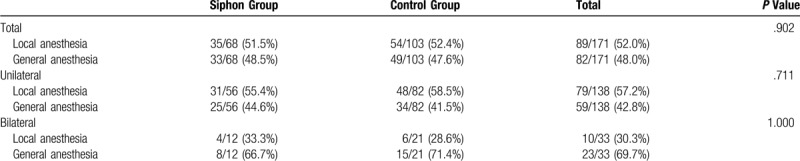
Anesthesia.

Table 4 lists the primary and secondary outcome measures. Compared with the control group, the siphon group presented with significantly better postoperative CT features, which included postoperative hematoma volume, hematoma evacuation rate, recovery rate of the midline shift, and the occurrence of pneumocephalus. In the siphon group, the length of hospital stay after the operation was 7 days (IQR: 6–9 days), which was significantly shorter than that of the control group (9 days; IQR: 6–11 days; *P* = .015). Although the recurrence rate in the siphon group (2/68, 2.5%) was lower than that in the control group (11/103, 8.9%), no statistically significant difference was observed between them (*P* = .069).

**Table 4:**
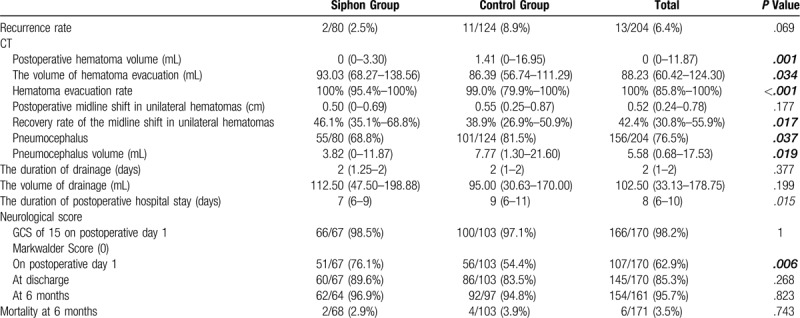
Primary and secondary outcome measures.

There was no significant difference between the two groups regarding mortality rate. One patient with a pulmonary infection in the siphon group had been sedated and intubated at the time he was transferred to our hospital; therefore, neurological function could not be evaluated. This patient died after surgery in the intensive care unit of our hospital due to complications from the pulmonary infection. Two patients in the control group died after discharge from a subdural hematoma recurrence that was not treated. One patient in the siphon group and two patients in the control group died after discharge, and the family members could not accurately describe the cause of death. We were, therefore, unable to conduct a six-month follow-up on the neurological function of the six patients who had died. Meanwhile, in the siphon group, two patients exhibited aggravated Parkinson's disease, which affected the neurological function score. In the control group, one patient with aggravated Parkinson's disease and another patient with a brain tumor could not be evaluated for neurological function. According to the comparison results, the Markwalder score of the siphon group was significantly superior to that of the control group on postoperative day 1, but not at discharge or 6 months after discharge.

No significant difference in complications was observed between the 2 groups (Table 5).

**Table 5:**
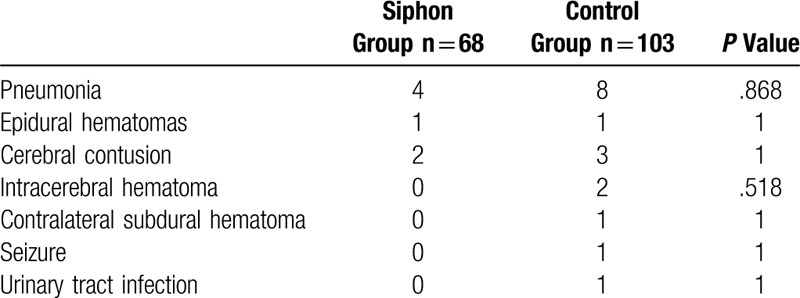
Postoperative complications.

## 4 Discussion

An international survey showed that among the various surgical treatments for the evacuation of CSDHs, BHC with a closed-system drainage is the preferred method.^[13]^ Intraoperative irrigation is an important part of this procedure, but multiple meta-analyses have shown that irrigation may be unnecessary as it may or may not affect the recurrence rate and the occurrence of complications.^[9,14,15]^ However, several studies have suggested that irrigation may lead to a better outcome, as it was assumed to reduce the number of coagulation factors (plasminogen activator) and inflammatory factors (interleukin-6 and vascular endothelial growth factor) in the hematoma fluid, which are associated with recurrence.^[16,17]^ To bring better outcomes for CDSH patients undergoing BHC, we tried to improve the effectiveness of irrigation by modifying the method. In this study, the siphon irrigation method demonstrated remarkable advantages in evacuating hematomas, recovering a midline shift, reducing the occurrence of pneumocephalus, improving neurological function, and reducing postoperative hospitalization.

### 4.1 Hematoma evacuation

The traditional method of irrigation involves pouring the irrigation fluid (usually normal saline) into the hematoma cavity through a catheter, and the liquefied hematoma is then evacuated from the cranial hole. The fluid discharged by intraoperative irrigation is a mixture of liquefied hematoma and irrigation fluid—also known as a diluted liquefied hematoma. The irrigation efficiency is low, especially in the later stage of the operation. After removing most of the hematoma, the dilution degree increases and the irrigation efficiency is further reduced. However, the siphon method, which uses the siphon principle to extract the hematoma, can evacuate a liquefied hematoma as a whole with higher efficiency and without being affected by the procedure.

On the other hand, to reduce the occurrence of pneumocephalus during the operation, the bone hole is positioned at the highest plane of the head; thus with the traditional method, the liquefied hematoma must be pushed out from the low position to the high position. The siphon method avoids the above situation by utilizing the siphon principle, which improves the irrigation efficiency.

**Figure 2:**
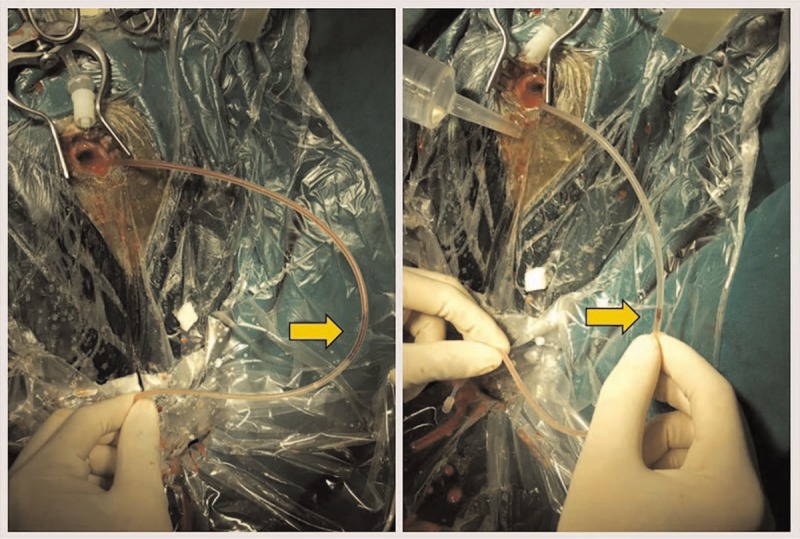
The orange arrow shows the non-liquefied hematoma fragments.

To effectively irrigate the hematoma cavity, several neurosurgeons have adopted the used of 2 burr holes during the operation.^[4,13]^ Although the hematoma can be evacuated completely, these procedures significantly increase the trauma to patients. By positioning the catheter in multiple directions, the siphon method is able to easily suction those deep hematomas and even non-liquefied hematoma fragments that are in the margin or far from the incision spot (Fig. 2). Therefore, to surgically manage a single hematoma without septa, one burr hole is required for adequate evacuation by siphon irrigation.

### 4.2 Midline shift

**Figure 3:**
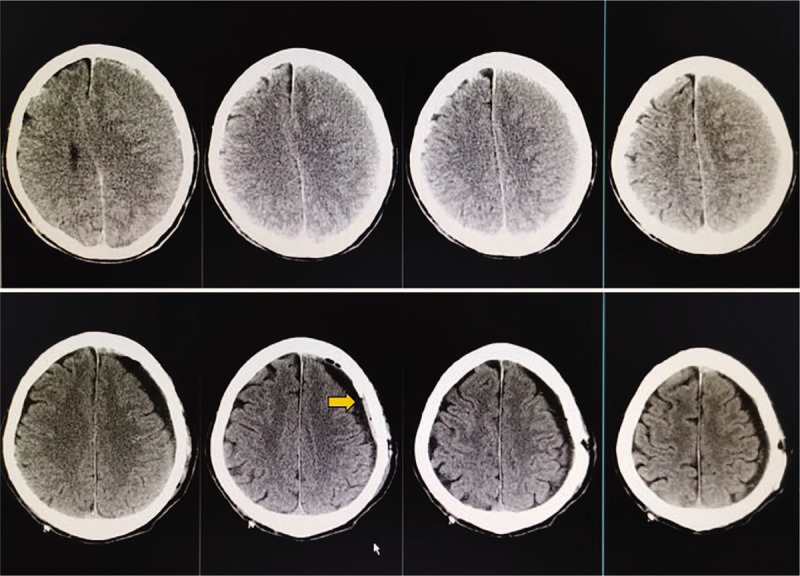
Upper: Preoperative CT. Lower: Postoperative CT. The orange arrow shows the fully expanded subarachnoid space, and the drainage tube inside the hematoma cavity is pushed against the skull.

Deficient re-expansion of the brain and insufficient recovery of the midline shift are considered to be two important factors that lead to hematoma recurrence.^[18,19]^ The traditional method of irrigation requires the injection of fluid into the hematoma cavity through the catheter. To achieve the effect of irrigation, a certain level of pressure must be applied, which inevitably affects the brain tissue; this is not conducive to brain tissue re-expansion. The siphon method utilizes pressure in the opposite direction. The suction effect does not affect the brain tissue, and may instead promote its re-expansion and the circulation of cerebrospinal fluid to a certain extent. We observed the expansion of the subarachnoid space in the postoperative CT scans of the siphon group (Fig. 3), but it was rarely observed in the control group.

Nevertheless, a more thorough removal of the hematoma results in better recovery of the midline shift.

### 4.3 Pneumocephalus

The space in the hematoma cavity changes constantly during the operation as a result of brain pulsation. Fluids are discharged when the tide rises and air will enter if it is not replenished when the tide falls; this contributes to the occurrence of pneumocephalus. In the siphon group, irrigation fluid was continuously injected into the hematoma cavity, which maintained the full volume of hematoma cavity and prevented gas entry while discharging the hematoma fluid. The traditional method intends to inject irrigation fluid into the hematoma cavity intermittently. During the interval of irrigating, the air is easily led into the hematoma cavity which would cause the pneumocephalus getting worse. Therefore, in this study, the siphon group was superior to the control group in the occurrence of pneumocephalus and pneumocephalus volume.

### 4.4 Neurological score and length of postoperative hospitalization

In this study, the neurological function score of the siphon group on the first day after surgery was significantly better than that of the control group. We believe that this result is attributed to a more efficient hematoma evacuation, better midline return, and lower pneumocephalus volume. Meanwhile, we were inclined to discharge the patients after recovery from serious neurological dysfunction; therefore, no statistically significant difference was observed in the neurological function scores at discharge, but the postoperative hospital stay of the siphon group was shorter and exhibited statistical significance. There was no significant difference in the neurological function score between the two groups at 6 months after surgery, suggesting that different irrigation methods had little effect on long-term neurological function recovery.

### 4.5 Recurrence rate

A considerable amount of literature has shown that the extent of hematoma evacuation,^[2–21]^ postoperative midline shift,^[22]^ and the occurrence of pneumocephalus^[23,24]^ are independent risk factors for the recurrence and reoperation of CSDHs. The siphon group performed better in these aspects, yet there was no significant statistical difference in the recurrence rate between the 2 groups.

### 4.6 Complications and safety

In the siphon group, the complications—particularly intracranial hemorrhage—were similar to those in the control group, and no statistical difference in mortality rate was noted between the 2 groups at 6 months after surgery. This finding suggests that siphon irrigation is a safe surgical method and is unlikely to cause new intracranial hemorrhage due to suction. The siphon effect is gentle, though it is inadequate when suctioning thick hematoma fluid, which should be diluted with a small amount of irrigation fluid for smooth evacuation in the early stage of the operation. The siphon function is stable, and the suction force can be easily adjusted by changing the tail height of the catheter. However, in any case, an extremely low drainage position and highly rapid drainage speed should be avoided during the procedure.

### 4.7 Limitations

This study features several limitations. First, some important indicators, such as operation duration, were not recorded. Second, given the limitation of conditions, BHC without irrigation was excluded from comparison. Most importantly, this research is a retrospective study. Randomized controlled studies based on large populations may be needed to further confirm these results in the future.

## 5 Conclusions

When compared with the traditional irrigation method in BHC, the siphon irrigation technique cannot significantly reduce the recurrence rate of CSDHs. However, the siphon irrigation technique is more effective in evacuating hematomas, improving the midline shift, and preventing the occurrence of pneumocephalus. Additionally, it is found to facilitate early recovery of neurological dysfunction after surgery and reduce the length of hospital stay. These findings suggest that siphon irrigation may be a better therapeutic option in BHC for CSDH.

## Acknowledgments

We would like to thank Dr. Hua-jun Chen, Dr. Zi-wen Zhao and Dr. Jia-bin Wang for their help with this study.

## Author contributions

Xian-kun Tu M.D. and Song Chen M.D. conceived and designed the study.

Bin Yang M.D. and Tao Xu M.D. were responsible for data collection.

Song Chen M.D. and hen Chen M.D. made major contributions to analysis and interpretation.

Song Chen M.D. was responsible for statistical analysis and drafting the article.

Xian-kun Tu M.D. and Zhen Chen M.D. critically revised the article.

All authors have reviewed submitted version of manuscript.

Xian-kun Tu M.D. and Zhen Chen M.D. performed administrative, technical and material support.

Xian-kun Tu M.D. was in charge of study supervision.
